# Validation of two complementary oral-health related quality of life indicators (OIDP and OSS 0-10 ) in two qualitatively distinct samples of the Spanish population

**DOI:** 10.1186/1477-7525-6-101

**Published:** 2008-11-18

**Authors:** J Montero, M Bravo, A Albaladejo

**Affiliations:** 1 Department of Surgery, School of Dentistry, University of Salamanca, Campus Unamuno, 37007, Salamanca, Spain; 2 Department of Public Dental Health, School of Dentistry, University of Granada, Campus de la Cartuja, 18071, Granada, Spain

## Abstract

**Background:**

Oral health-related quality of life can be assessed positively, by measuring satisfaction with mouth, or negatively, by measuring oral impact on the performance of daily activities. The study objective was to validate two complementary indicators, i.e., the OIDP (Oral Impacts on Daily Performances) and Oral Satisfaction 0–10 Scale (OSS), in two qualitatively different socio-demographic samples of the Spanish adult population, and to analyse the factors affecting both perspectives of well-being.

**Methods:**

A cross-sectional study was performed, recruiting a Validation Sample from randomly selected Health Centres in Granada (Spain), representing the general population (n = 253), and a Working Sample (n = 561) randomly selected from active Regional Government staff, i.e., representing the more privileged end of the socio-demographic spectrum of this reference population. All participants were examined according to WHO methodology and completed an in-person interview on their oral impacts and oral satisfaction using the OIDP and OSS 0–10 respectively. The reliability and validity of the two indicators were assessed. An alternative method of describing the causes of oral impacts is presented.

**Results:**

The reliability coefficient (Cronbach's alpha) of the OIDP was above the recommended 0.7 threshold in both Validation and Occupational samples (0.79 and 0.71 respectively). Test-retest analysis confirmed the external reliability of the OSS (Intraclass Correlation Coefficient, 0.89; p < 0.001) Some subjective factors (perceived need for dental treatment, complaints about mouth and intermediate impacts) were strongly associated with both indicators, supporting their construct and criterion validity. The main cause of oral impact was dental pain. Several socio-demographic, behavioural and clinical variables were identified as modulating factors.

**Conclusion:**

OIDP and OSS are valid and reliable subjective measures of oral impacts and oral satisfaction, respectively, in an adult Spanish population. Exploring simultaneously these issues may provide useful insights into how satisfaction and impact on well-being are constructed.

## Background

According to the World Health Organization [1], evaluation of the health of subjects requires assessment of their physical, psychological and emotional well-being, not merely confirmation of disease absence. Thus, measurement of the impact of oral conditions on quality of life should be part of the evaluation of oral health needs. Clinical indicators alone cannot describe the satisfaction or symptoms of dental patients or their ability to perform daily activities.

Over the past three decades, questionnaires and scales have been developed to reflect the impact of oral diseases on the daily activities of individuals. This information complements clinical data to describe the oral health-related quality of life (OHRQoL). There are no universally accepted definitions of OHRQoL or of its dimensions or the main factors involved, which vary among different social, cultural and political settings, as reported by Locker [2].

There is a growing trend to utilise and compare a small number of OHRQoL indicators across different cultures in order to achieve cross-cultural validation. Thus, a European project [3] recommended focussing on three OHRQoL indicators: OHIP-14 [4], OHQoL-UK [5] and OIDP [6]. The OIDP (Oral Impacts on Daily Performances) is a commonly used OHRQoL indicator that assesses the impact of oral conditions on the individual's abilities to perform daily activities linking the causal entities involved. The OIDP has been shown to have adequate psychometric properties in different populations [6-16] proving to be reliable and valid in cross-sectional population-based studies.

Prior to the development of the OHRQoL indicators subjective perceptions of oral health were usually gathered by means of single-item global indicators. These apparently simple measures continue to be widely used in quality of life research. A simple oral satisfaction scale (OSS) has already been successfully used in cross-sectional and longitudinal studies [17] as a unidimensional indicator of oral well-being.

Oral well-being can be comprehensively evaluated by the simultaneous application of indicators of oral impacts (OIDP) and oral satisfaction (OSS), because both could be considered major and complementary dimensions of OHRQoL.

As the psychometric properties of scales must be re-evaluated when used in a new population [18] and the OHRQoL could be directly or indirectly affected by the socio-economic status [19], the main objective of this study was to validate OIDP and OSS in two qualitatively different socio-demographic samples of the Spanish adult population, evaluating the OHRQoL by using both an "impact" and a "satisfaction" approach.

## Methods

### Oral impacts on daily performances

The Oral Impacts on Daily Performances index (OIDP) is an intuitive OHRQoL indicator that focuses solely on the impact on the individual's performance of daily activities. The OIDP [6] is inspired by a theoretical model developed by the World Health Organization [20] and adapted for oral health by Locker [21], differing in its division of the consequences of oral conditions into *impairments*, i.e., structural or functional disturbance of stomatognatic system; *intermediate impacts*, i.e., pain, discomfort, functional limitation and dissatisfaction with appearance; and *ultimate impacts*, equivalent to disability and handicap dimensions in the WHO model [20]. The OIDP only takes into account the frequency and perceived severity of the *ultimate impacts*, thereby minimising possible over-scoring of the index.

The first level (*impairments*) refers to the immediate biophysical outcomes of disease, which most clinical indices attempt to evaluate, whereas the *intermediate *and *ultimate *impacts can only be assessed by the individuals themselves. For an *impairment *to have *ultimate *impact, the pain, discomfort, functional limitation or dissatisfaction with appearance must be perceived as affecting the individual's physical, psychological or social performance. In the OIDP index impacts are quantified by multiplying the frequency and severity scores to obtain the performance score for each of eight dimensions. The sum of these scores is considered the total impact score. This total score is divided by the maximum possible score and multiplied by 100 to give the percentage score. This scoring system yields an intuitive oral impact score. The frequency and severity scores are Likert-type scales, but a zero score is only possible for severity. Hence, severity is weighted and can produce a zero score for an impact if the individual considers that there is no effect on daily life activities. This scoring method, which was used by Leao and Sheiham in Dental Impacts on Daily Living [22], an earlier indicator from the same research team, is coherent with the aforementioned theoretical base and with the current consensus on the assessment of perceptions.

For each dimension (eating, speaking, cleaning teeth, working, social relation, sleeping/relaxing, smiling and emotional status), the oral or dental condition that caused the most severe impact were recorded. In order to analyse the relative burden of impacts among dimensions, three intuitive descriptors of the causes of impacts were used: "*impact value*", i.e., number of impacts generating a given causal entity, regardless of the dimensions they were recorded in; "*impact extension*", i.e., the number of dimensions affected by a given causal entity; and "*impact prominence*", i.e., the percentage of impacts attributable to a given causal entity in a given dimension.

### Oral satisfaction assessment

The Oral Satisfaction Scale (OSS) is a visual analogue scale (0 to 10) that allows subjects to weigh their perceived oral satisfaction. Measuring self-assessment of oral satisfaction is an attractive method to evaluate the OHRQoL, because it allows respondents to evaluate their own specific dimensions in the process of quantifying their perceived level of satisfaction. McDowell and Newell [23] claimed that individuals can make subjective judgements in a reliable manner if well-demarcated ordinal scales are used. The 0–10 scale has been widely used as a gold standard to assess oral health status in cross-sectional [24] and longitudinal studies [25]. OSS is defined as a measure of psychological well-being in relation with mouth. It was hypothesized that oral satisfaction should be affected by clinical conditions disrupting the individual's physical, psychological or social performance (as the OIDP), but also some non impact-related factors, such as present and past values, expectations and beliefs, could variously impinge on that feeling.

### Validation process

The process of developing and evaluating the OIDP and the OSS for the Spanish population consisted of three main steps: linguistic and cultural adaptation of the original OIDP to the Spanish setting using the back-translation method [26]; pilot study to assess face and content validity; and main study to assess the reliability and construct validity in two distinct socio-demographic samples of the Spanish population.

The psychometric properties of an instrument for measuring perceptions must be tested by evaluating its reliability and its validity [18]. In multidimensional instruments such as the OIDP, the reliability is evaluated by testing the internal consistency or homogeneity of the scale, i.e., different dimensions of the instrument evaluate distinct aspects of the same attribute [27]. In unidimensional scales such as the OSS, the reliability must be objectively supported by Test-retest analysis to show stability over time. Both instruments were also assessed for face, content, criterion, construct and convergent validities.

### Linguistic and cultural adaptation

Because the OIDP and OSS had not previously been used in Spain, the Spanish version of these instruments were piloted to assess their face and content validity in this population. The OIDP and OSS were linguistically and culturally adapted to our setting by using the back translation technique [26]. In this procedure, translations were independently made by two bilingual dentists, who then discussed and produced a consensus Spanish version, which was translated back into English by a professional English native translator who had not seen the original version. The conceptual equivalence between the original instruments and the back-translated versions was supported by an expert committee (formed by 5 university researchers on quality of life studies). The definitive Spanish version was produced after the face and content validity results in the pilot study had been approved by this committee.

### Pilot study

Ethical approval was obtained from the relevant authorities (Bioethics Committee of the University of Granada, Health Districts and the Employment Risk Prevention Centre) before the pilot and main studies were started. All participants were briefed about the purpose and process of the study and filled the explicit written consent. The pilot study was conducted in a convenience sample (n = 54) recruited from among dental patients coming to the School of Dentistry for a check-up and their companions. The 54 participants were clinically examined and interviewed, using the pilot versions of the two indicators. The comprehensiveness of the indicators was tested by detecting and asking questions on difficulties in understanding items, scales or the content of the dimensions, in order to improve the intelligibility of the instruments when necessary and optimise the face and content validity for the main study.

### Main study

A cross-sectional epidemiological study was performed in Granada capital and province. In order to validate the indicators in two distinct socio-economic groups, two types of samples were recruited: a sample of the general population, designated "Validation Sample"; and a sample of the healthy employed population, designated "Working Sample". Age < 25 years was an exclusion criterion, since OIDP and OSS were originally designed for adults, and individuals seeking dental treatment were also excluded in order to establish baseline impact scores for the Spanish population.

The Validation Sample (n = 253) was recruited from among non-dental patients and their companions at three randomly selected Heath Centres in the City and Metropolitan Health Districts of Granada. This sample was used for a preliminary validation study of OIDP and OSS, for which a sample size of 100–200 is recommended [17]. The Working Sample (n = 561) was recruited from among healthy Andalusia Regional Government staff visiting the Employment Risk Prevention Centre for a routine medical check-up. All interviewees were briefed about the purpose and process of the study and consent was obtained for questionnaire-led interviews and simple oral examination.

Socio-demographic (age, gender, occupation), behavioural (e.g., toothbrushing frequency, dental visits) and clinical (e.g., presence of caries, periodontal disease and prosthesis) data were collected from all participants. Impacts on quality of life were gathered by using the piloted OIDP and the satisfaction level was assessed by the OSS. Oral examinations were performed by an examiner calibrated for the criteria established in the 1987 WHO dossier [28], which were used by the most recent Oral Health National Survey in Spain. The interview was conducted by an examiner trained in the theoretical postulates of OIDP and OSS.

Because there is no universally accepted gold standard for assessing criterion validity of quality of life measures and a key property of these instruments is their contribution to needs assessment, data were collected on perceived treatment needs as a proxy. Construct validity was evaluated by testing the outcomes of the OIDP and OSS against complaints about the mouth, considered as a proxy of the intermediate or perceived impairment in accordance with the theoretical framework. After the reliability of the OSS had been confirmed in the pilot study by test-retest, it was also used as a proxy to test the convergent validity of the OIDP. It was predicted that oral impacts on daily performances (OIDP) would negatively affect oral satisfaction (OSS).

In the Working Sample, the most highly valued aspects of the mouth and intermediate impacts were also recorded to assess the adequacy of the OIDP to capture the perceptions of individuals.

### Statistical analysis

The Statistical Package for Social Sciences v.13. (SPSS Inc., Chicago, IL) was used for the statistical analyses. The cut-off level for statistical significance was 0.05. The internal consistency of the OIDP was assessed by standardised Cronbach's alpha, Cronbach's alpha-if-item-deleted, inter-item and item-total correlation coefficients. As the OIDP total scores were not normally distributed and because some groups comparisons undertaken involved relatively small cell sizes, tests for criterion and construct validity were non-parametric (Mann-Whitney and Kruskal-Wallis Test as appropriate). The modulating factors were explored by using both Pearson (r) and Spearman (r_s_) correlation coefficients. Test-Retest Reliability of the OSS was evaluated with the Intraclass Correlation Coefficient (ICC).

## Resuts

### Pilot study

The fact that the ODIP independently gathers the frequency score, severity score and perceived cause of impact was considered sufficient by the expert committee to verify its face validity. The content validity was also considered satisfactory since it included oral health-related dimensions (eating, speaking, cleaning...) and physical, psychological and social dimensions related to daily life activities. The adequacy of the OSS, designed as a visual analogue scale, was also approved by the expert committee for use as a simple unidimensional measure of the degree of oral satisfaction, which is believed to range across a continuum of values. Moreover, while the OIDP only assess negative oral experiences, the OSS is a bidirectional measure of oral satisfaction, being able to measure either positive or bad feelings. Face and content validities were confirmed in the pilot study, since no misunderstanding of any item or scale was detected in or reported by the 54 participants. Only 3 subjects (5.5%) reported that OIDP missed a dimension of oral function (all referred to a sexual function). Test-retest reliability ensured that all subjects were self-designated as satisfied (score > 5), neutral (score = 5) or dissatisfied (score < 5) in a consistent way, although there was a small variation in scores for satisfied and dissatisfied (ICC: 0.87; p < 0.001).

### Validation sample

A total of 280 individuals were invited to participate in the Validation Sample and 253 (90.4%) accepted. The mean age was 55.9 ± 16 years, 39.5% were male, 56.5% belonged to a low occupational class, > 75% brushed their teeth at least once a day and > 80% had visited the dentist at least once in the previous 5 years.

Validation Sample participants had a mean of 3.4 ± 4.7 replaceable teeth, and 68.8% were dentate without removable prostheses. They had a mean of 14.2 ± 8.1 healthy non-restored teeth and a DMFT index score of 14.4 ± 7.4 (3.6 ± 3.2 decayed, 8.5 ± 8.7 missing and 2.3 ± 2.8 filled teeth). The Community Periodontal Index score was zero in 1.7 ± 2.0 of sextants.

The internal consistency or homogeneity of the OIDP was tested by analysing the matrix of correlations among items and confirming the absence of negative correlations or variations in magnitude that were large enough for an item to be considered redundant. The inter-item correlation coefficients between scores of the 8 dimensions ranged from 0.10 (between Cleaning and Working) to 0.62 (between Social and Smiling). A search for weighted items was then conducted by analysing the correlation of each item with the total OIDP score, finding that all correlations were > 0.20 (Table 1). The standardised Cronbach's alpha value obtained from the correlation matrix was 0.79, and this alpha value was not increased by the removal of any item. In fact, the removal of some items lowered this value, further supporting the inclusion of all of the original items.

**Table 1:** Reliability test of OIDP among the validation sample (n:253).

OIDP Dimensions	Corrected item-total correlation	Alpha if item deleted
Eating	0.46	0.77
Speaking	0.57	0.75
Cleaning	0.38	0.78
Working	0.36	0.78
Social	0.57	0.75
Sleeping & Relaxing	0.47	0.77
Smiling	0.53	0.75
Emotional	0.68	0.73

Analysis of corrected item-total correlation and Alpha value if item deleted.

Alpha = 0.78

Standardised item Alpha = 0.79

Criterion validity was assessed by using a single-item assessment of perceived treatment need (Table 2). Individuals who reported dental treatment need in the validation sample obtained a significantly higher OIDP score and lower OSS score compared with those perceiving no treatment need. With respect to the construct validity, the mean total OIDP score was significantly lower in those with no complaints about the mouth than in those with complaints and their self-rated satisfaction was significantly higher. Regarding convergent validity, the OIDP score was significantly lower in the satisfied than in the neutral or dissatisfied groups. The OSS scores showed the expected inverse relationship with OIDP scores (r = -0.44, p < 0.01).

**Table 2:** Validity test for OIDP and OSS among the validation sample (n = 253).

	n (%)	OIDP 95% CI	OSS 95% CI
	CRITERION VALIDITY		

PERCEIVED TREATMENT NEEDS			
NO	104 (41.1%)	1.9 – 5.1	8.5 – 8.8
YES	149 (58.9%)	10.2 – 17.0	4.8–5.5
t		p < 0.001	p < 0.001

	CONSTRUCT VALIDITY		
PERCEIVED ORAL WELL-BEING			
No complaint	38 (15%)	0.2–1.6	8.0–8.7
With complaint	215 (85%)	8.6–13.8	5.9–6.6
		p < 0.001	p < 0.001

	CONVERGENT VALIDITY		
ORAL SATISFACTION			
< 5 (DISSATISFIED)	41 (16.2%)	12.9–26.0	2.5–3.5
5 (NEUTRAL)	48 (19.0%)	10.1–25.2	No sense
> 5 (SATISFIED)	164 (64.8%)	2.9–6.0	7.8–8.2
		p < 0.001	p < 0.001

Mann-Witney Test for "Perceived Treatment Needs" and "Perceived Oral Well-being".

Kruskal-Wallis Test for "Oral Satisfaction"

95% CI = 95% Confidence Interval

As depicted in Table 3, the OIDP and OSS demonstrated significant (p < 0.05) correlation with socio-demographic, behavioural and clinical variables, allowing the identification of modulating factors. Among socio-demographic variables, there were highly significant differences in OIDP score between the sexes, with females showing a higher level of impact versus males. Main behavioural findings were that a greater satisfaction was associated with higher tooth brushing frequency and a greater impact was associated with a longer period since a visit to the dentist. Among clinical variables, impact and satisfaction levels were influenced by dental caries data, e.g., number of teeth with caries, and this correlation was stronger when only visible (interpremolar) teeth were considered. Some prosthetic variables influenced the impact level (i.e. number of occlusal units) and others the satisfaction level (i.e., number of absent teeth replaced). No periodontal variables were significantly associated with oral impacts, but the number of sextants with dental mobility was highly correlated with satisfaction.

**Table 3:** Modulating factors of OIDP and OSS among the validation sample (n = 253).

**SOCIO-DEMOGRAPHIC VARIABLES**	OIDP	OSS
Gender		
Male (mean ± sd)	6.3 ± 12.1**	6.7 ± 2.3
Female (mean ± sd)	11.3 ± 16.6**	6.5 ± 2.2

**BEHAVIOURAL VARIABLES**		
Last previous visit to dentist	r_s _= -0.1*	r_s _= 0.06
Tooth brushing frequency	r_s _= 0.05	r_s _= - 0.16*

**PROSTHODONTIC VARIABLES**		
Normative Needs for prosthesis	r_s _= 0.15*	r_s _= -0.18*
No occlusal units	r = - 0.14*	r = 0.05
No aesthetic units	r = - 0.03	r = 0.06
No replaceable absent teeth	r = 0.17**	r = - 0.20**
No replaced absent teeth	r = - 0.05	r = 0.18*
No replaceable visible teeth	r = 0.16**	r = - 0.20**
No replaced visible teeth	r = 0.05	r = 0.18*
No replaceable functional teeth	r = 0.15*	r = -0.17*

**CARIES VARIABLES**		
No teeth with caries requiring endodontic treatment	r = 0.24**	r = -0.24**
No teeth with caries requiring extraction	r = 0.18**	r = -0.14
No teeth with 2 or more decayed surfaces	r = 0.18**	r = -0.17*
No teeth with caries	r = 0.22**	r = -0.19**
No visible teeth with caries	r = 0.33**	r = -0.28**
No healthy filled teeth	r = 0.02	r = -0.20**
No healthy restored visible teeth	r = -0.04	r = -0.22**
Decayed Missing and Filled Teeth (DMFT) Index	r = 0.13*	r = -0.06
Need for restorative treatment	r_s _= 0.20*	r_s _= -0.20*

**PERIODONTAL VARIABLES**		
No sextants with CPITN score of 1	r = -0.08	r = 0.03
No sextants with dental mobility	r = 0.10	r = -0.27*

Mann-Whitney Test for Gender. Correlation for the remainder (r = Pearson correlation; r_s _= Spearman correlation)

*p < 0.05; ** p < 0.01

### Working sample

The Working Sample comprised 561 healthy individuals who were all Regional Government staff, presumed to represent the more privileged end of the socio-demographic spectrum of the reference population. The mean age was 43.2 ± 8.8 years, 46.5% belonged to middle occupational class, and 51.9% were females. Teeth were brushed once or twice a day by 60% of the sample and three times a day by 32.5%. Programmed visits to the dentist were made at least every two years by 54.5% of this sample, while the remainder made visits when they experienced oral problems.

Working Sample showed a good state of oral health. More than 90% were dentate without removable prostheses. They had a mean of 17.8 ± 5.8 healthy non-restored teeth and a DMFT index score of 11.0 ± 5.1, with a Community Periodontal Index score of zero in 3.2 ± 2.2 of sextants.

As in the general population, the OIDP again demonstrated its internal consistency in the correlation matrix, with no negative correlations or redundant items. The inter-item correlations ranged from 0.10 (Cleaning-Smiling) to 0.48 (Social-Smiling). Item-total correlations showed that all items were above 0.20 and that the elimination of items reduced the Cronbach's alpha (Table 4). The standardised Alpha was 0.71.

**Table 4:** Reliability test of OIDP among the working sample (n:561).

OIDP Dimensions	Corrected item-total correlation	Alpha if item deleted
Eating	0.43	0.65
Speaking	0.40	0.67
Cleaning	0.30	0.68
Working	0.38	0.67
Social	0.43	0.66
Sleeping & Relaxing	0.38	0.67
Smiling	0.42	0.66
Emotional	0.45	0.65

Analysis of corrected item-total correlation and Alpha value if item deleted.

Standardised item Alpha = 0.71

Alpha = 0.69

Regarding the criterion validity (Table 5), individuals who perceived need for dental treatment had much higher OIDP and lower OSS in comparison to those that did not (p < 0.001). With respect to the construct validity, individuals reporting a mouth-related complaint or an intermediate impact scored significantly higher in the OIDP (greater ultimate impact) and significantly lower in the OSS (lower oral satisfaction). The convergent validity was confirmed by the coherent inverse relationship of indicators in relation to each other (r = - 0.42; p < 0.01).

**Table 5:** Validity test for OIDP and OSS among the working sample (n = 561).

	n (%)	OIDP 95% CI	OSS 95% CI
	CRITERION VALIDITY		

PERCEIVED TREATMENT NEEDS			
No	251 (44.7%)	2.1 – 4.4	6.9 – 7.4
Yes	310 (55.3%)	8.4 – 13.4	5.5–6.3
		p < 0.001	p < 0.001

	CONSTRUCT VALIDITY		

PERCEIVED ORAL WELL-BEING			
No complaint	160 (28.6%)	1.2–2.4	7.5–7.9
With complaint	401 (71.4%)	6.2–8.5	6.0–6.3
		p < 0.001	p < 0.001

INTERMEDIATE IMPACTS			
None	160 (28.6%)	1.2–2.4	7.5–7.9
Appearance	122 (21.8%)	3.7–7.4	5.9–6.6
Susceptibility	95 (16.9%)	3.4–8.3	5.7–6.5
Pain	76 (13.5%)	5.5–10.4	6.0–6.8
Functional limitation	70 (12.5%)	6.6–12.1	5.2–6.1
Discomfort	27 (4.8%)	7.4–18.8	5.7–6.9
Others	11(1.9%)	0.7–14.3	5.2–8.2
		p < 0.001	p < 0.001

	CONVERGENT VALIDITY		

ORAL SATISFACTION			
< 5 (Dissatisfied)	74 (13.2%)	13.1–20.1	3.3–3.7
5 (Neutral)	73 (13.0%)	4.2–7.9	No sense
> 5 (Satisfied)	414 (73.8%)	2.9–4.5	7.3–7.5
		p < 0.001	p < 0.001

Mann-Witney Test for "Perceived Dental Need" and "Perceived Oral Well-being".

Kruskal-Wallis Test for "Intermediate Impacts" and "Oral Satisfaction"

In the Working sample, some observations were made to ensure the suitability of the dimensional battery of the OIDP for the target population. The most highly valued aspects of the mouth were disease-free (27.5%), appearance (27.3%), eating (19.4%), cleaning (13.9%), odour (7.7%), pain-free (3%) and other aspects (1.2%). Moreover the intermediate impacts obtained by an open response question on the main mouth-related complaint could be matched with those in the theoretical model, with the exception of "susceptibility" to oral disease, which had not previously been reported (Table 5). The most prevalent intermediate impacts were dissatisfaction with appearance (21.8%), pain (13.5%), functional limitation (12.5%) and discomfort (4.8%).

Moreover, the last 269 participants of the Working Sample were asked about the influence of the mouth on their occupational performance, and 168 (62.5%) believed that their mouth could affect their work, citing the following causes: dental pain (64.7%), appearance (15.0%), speaking (12.6%) and mouth odour (7.7%).

Modulating factors were established by correlations with socio-demographic, behavioural and clinical variables (Table 6). Females reported a higher level of impact (OIDP score) and lower satisfaction (OSS score) compared with males. Among clinical conditions, caries factors influenced impact and satisfaction levels, whereas prosthodontic variables were significantly associated with satisfaction but not impact levels. Among periodontal variables, a good state of periodontal health was associated with greater satisfaction but not with impact; but a bad state of periodontal health with dental mobility was associated with both indicators.

**Table 6:** Modulating factors of OIDP and OSS among the working sample (n = 561).

**SOCIODEMOGRAPHIC VARIABLES**		OIDP	OSS
Gender			
Male (mean ± sd)		3.8 ± 7.7***	6.8 ± 1.7*
Female(mean ± sd)		6.9 ± 11.4***	6.4 ± 1.8*

**BEHAVIOURAL VARIABLES**			
Last previous visit to dentist		r_s _= - 0.1**	r_s _= 0.06

**PROSTHODONTIC VARIABLES**			
Normative Needs for prosthesis		r_s _= 0.04	r_s _= -0.23**
Type of edentulism (Eichner Index)		r_s _= 0.08	r_s _= -0.28**
No occlusal units		r = - 0.06	r = 0.27**
No aesthetic units		r = - 0.03	r = 0.12**
No absent teeth		r = 0.04	r = -0.22**
No replaceable absent teeth		r = 0.07	r = -0.23**
No replaced absent teeth		r = - 0.01	r = -0.11*
No replaceable visible teeth		r = 0.01	r = -0.14**
No replaceable functional teeth		r = 0.07	r = -0.25**
No replaced visible teeth (Fixed or Removable Prothesis)		r = - 0.01	r = -0.10*
No replaced functional teeth (Fixed or Removable Prothesis)		r = - 0.04	r = -0.10*
No natural teeth present		r = - 0.02	r = 0.21**
Prosthetic groups			
Dentate without prosthesis (mean ± sd)		5.4 ± 10.0	6.6 ± 1.7 *
Wearers of removable prosthesis (mean ± sd)		5.4 ± 8.9	6.1 ± 2.2*

**CARIES VARIABLES**			
No healthy unfilled teeth		r = -0.04	r = 0.26**
No teeth with caries requiring extraction		r = 0.17**	r = -0.10*
No visible teeth with caries		r = 0.11**	r = -0.15**
No healthy filled teeth		r = -0.02	r = 0.09*
No healthy filled visible teeth		r = -0.04	r = -0.15**
Decayed Missing and Filled Teeth (DMFT) Index		r = 0.04	r = -0.27**
Need for restorative treatment		r_s _= 0.1*	r_s _= 0.1*

**PERIODONTAL VARIABLES**			
No sextants with CPITN score of 0		r = -0.08	r = 0.13**

No sextants with dental mobility =	0	r = -0.09*	r = 0.15**
	1	r = 0.02	r = -0.03
	2	r = 0.11**	r = -0.10*
	3	r = 0.07	r = -0.11*

Mann-Whitney Test for "Gender" and prosthetic groups. Correlation for the remainder (r = Pearson; r_s _= Spearman)

* p < 0.05; ** p < 0.01; ***p < 0.001

Table 7 depicts the distribution of causal entities reported by the Working Sample in each OIDP dimension. "Dental pain" was perceived to have the greatest effect on oral well-being (*impact value *= 80). "Third-molar pain" was considered separately due to its distinct symptoms and treatment approach. Both pain-related entities were wide-reaching variables that affected all dimensions except "Smiling" (*impact extension*). "Working" was the dimension most affected by dental pain and third-molar pain, which caused 31.3% and 12.5%, respectively, of recorded impacts (*impact prominence*), followed by "Eating dimension", for which the corresponding percentages were 28.0% and 6.1%.

**Table 7:** Percentage distribution of main causes of impact in working sample (n = 561)

DIMENSION CAUSE	**Eating**	**Speaking**	**Cleaning**	**Working**	**Social**	**Sleeping & Relaxing**	**Smiling**	**Emotional state**	Value
Oral ulcers.	3 (2.3%)	4 (21.1%)	1 (1.3%)	1 (6.3%)	1 (1.4%)	2 (2.5%)	1 (2.0%)	3 (5.6%)	16
Dental pain	37 (28.0%)	1 (5.3%)	6 (7.6%)	5 (31.3%)	2 (2.9%)	16 (19.8%)		13 (24.1%)	80
Third-molar pain	8 (6.1%)	1 (5.3%)	3 (3.8%)	2 (12.5%)	3 (4.3%)	4 (5.0%)		4 (7.4%)	25
Prosthesis	10 (7.5%)	3 (15.8%)			2 (2.8%)	2 (2.5%)	3 (5.9%)	2 (3.7%)	22
TMJ pain- dysfunction	6 (4.5%)			1 (6.3%)	1 (1.4%)	41 (50.6%)		6 (11.1%)	65
Missing teeth	9 (6.8%)	5 (26.3%)			6 (8.6%)		17 (33.3%)		37
Dental appearance					9 (12.8)	2 (2.5%)	16 (31.4%)	3 (5.6%)	30
Bad Breath				3 (18.8%)	38 (54.3%)			8 (14.8%)	49
Dental sensitivity	23 (17.4%)		18 (22.8%)					2 (3.7%)	43
Food Packing	18 (13.6%)		9 (11.4%)		3 (4.3%)				30
Gingival bleeding			26 (32.9%)						26
Other causes	18 (13.6%)	5 (26.3%)	16 (20.3%)	4 (24.8%)	5 (7.1)	14 (17.1%)	14 (27.4%)	13 (24.1%)	89
TOTAL n (% of sample))	132 (23.5%)	19 (3.4%)	79 (14.1%)	16 (2.9%)	70 (12.5%)	81 (14.4%)	51 (9,1%)	54 (9.6%)	

The most "*extensive*" impact was produced by "Oral ulcers", although their *impact prominence *and *impact value *were low. The least "*extensive*" impact was from "bleeding gums", which affected only the "cleaning dimension" but had an "*impact prominence*" of 32.9%.

"Bad breath" was the most prominent entity, accounting for 54.3% of impacts reported in the "Social dimension", followed by "TMJ pain-dysfunction", which caused 50.6% of impacts in the "Sleeping and Relaxing" dimension.

### Oral health-related quality of life

Once OIDP and OSS were found to satisfactorily meet validation criteria, the levels of impact and satisfaction recorded in our series were documented (Table 8). The prevalence of oral impacts was 58.1% in the Validation Sample versus 46.0% in the Working Sample, with mean total scores of 9.1 ± 14.8 and 5.7 ± 10.2, respectively. In both samples, the most frequently and most severely affected dimension was "eating" (38.3% and 23.5% respectively) and the least frequently and severely affected dimension was "working" (2.0% and 2.9% respectively). However the majority of individuals in both Validation and Working samples were satisfied with their mouth (64.7% and 73.8%, respectively).

**Table 8:** Prevalence of impacts (OIDP) and satisfaction (OSS) among the "validation" (n=253) and "working"(n=561) samples.

	VALIDATION SAMPLE		WORKING SAMPLE	
	
	[(n (%)]	Mean ± sd	[(n (%)]	Mean ± sd
Prevalence of impacts (OIDP)				
Some impact	147 (58.1%)	9.1 ± 14.8	248 (46.0%)	5.7 ± 10.2
Eating	97 (38.3%)	5.2 ± 7.6	132 (23.5%)	2.7 ± 5.6
Speaking	32 (12.6%)	1.7 ± 5.0	19 (3.4%)	0.4 ± 2.5
Cleaning	63 (24.9%)	2.8 ± 6.0	79 (14.1%)	1.4 ± 4.1
Working	5 (2%)	0.3 ± 2.3	16 (2.9%)	0.4 ± 2.8
Social	33 (13%)	2.2 ± 6.3	70 (12.5%)	1.9 ± 5.6
Sleeping & Relaxing	35 (13.8%)	2.5 ± 6.7	81 (14.4%)	1.9 ± 5.0
Smiling	28 (11.1%)	1.8 ± 5.7	51 (9.1%)	1.3 ± 4.6
Emotional	21 (8.3%)	1.7 ± 5.9	54 (9.6%)	1.4 ± 4.6

Prevalence of satisfaction (OSS)				
Dissatisfied	41 (16.3%)	3.0 ± 1.4	74 (13.2%)	3.5 ± 0.9
Satisfied	164 (64.7%)	8.0 ± 1.2	414 (73.8%)	7.4 ± 1.1

## Discussion

This study evaluates the validity of a multidimensional indicator of oral impacts (OIDP) and a unidimensional scale of oral satisfaction (OSS) applied simultaneously for assessing the oral well-being from those distinct but complementary perspectives. The population sample for this study was initially recruited from among non-dental patients and companions at Health Centres, considered a suitable approach for sampling the general population by the Andalusian Department of Epidemiology and Public Health. However, since recruitment was carried out during working hours, there was a bias towards low socio-occupational groups (e.g., pensioners and unemployed). Nevertheless, the wide age range of the sample and the exclusion of individuals seeking dental treatment yielded a valuable but preliminary validation of the indicators and estimation of the baseline impacts. Because socio-economic conditions might influence OHRQoL directly and indirectly [19], we recruited from among healthy active Regional Government officers to obtain another sample of the same reference population with a qualitatively higher socio-demographic profile.

Cross-cultural adaptation procedures are a critical component of the validation of an instrument developed in a different target population. In the present study, the translation to Spanish posed no difficulties, and comparison between the original OIDP and the back-translated English version revealed no conceptual or content differences. Equivalent words were readily found thanks to the simple structure of the original OIDP and the universal nature of its dimensions. On the other hand, it proved more challenging to comprehend the theoretical basis of the OIDP and its approach to quality of life measurements. The OSS was easier to adapt because of its formal simplicity. It is really not known what underlies expressions of satisfaction or dissatisfaction with mouth, but it is believed to be a measure of psychological well-being modulated by clinical conditions disrupting the individual's physical, psychological or social performance, and also by some non impact-related factors, such as present and past values, expectations, and beliefs, that have not been addressed in this study. We have found some modulating factors (mostly prosthetic variables) that impinged on satisfaction without altering the physical, psychological or social performances. Thus future research must be directed towards those potential non impact-related factors.

This study is the first to use the OIDP index in a Spanish population and the first OHRQoL study in Spanish adults. Both instruments (OIDP and OSS) proved to be valid and reliable indicators. Face and content validity were established in our pilot study by asking participants about the comprehensiveness of the instruments, which had already been approved by a panel of experts. The only method used to assure the understanding of older adults relied upon the communicating abilities of the examiner to adapt the container without altering the content. Moreover, the visual analogue scale used for the OSS was worded in some cases to show the conceptual equivalence and allow respondents to make appropriate self-ratings.

In both samples construct and criterion validity was demonstrated in that the OIDP and OSS scores discriminated in the expected direction between subjects who perceived dental treatment need or complaints about the mouth (Table 2 and 5). With regard to the convergent validity, the indicators showed a coherent and significant inverse relationship to each other (correlations coefficients ranging between -0.44 and -0.42 in Validation and Working samples respectively) and in relation with other subjective variables (Tables 2 and 5), supporting the study hypothesis that oral impacts and oral satisfaction are opposing but complementary approaches to the evaluation of oral well-being. It is plausible that OSS may be recognized as a proxy gold standard measure for OHQOL indicators since it is a simple but a powerful and discriminative measure.

Some authors have validated quality of life indicators by using subjective criteria but not clinical indicators [7-16,29-31] arguing that the latter evaluate disease states whereas quality of life indicators include psychological and sociological aspects that only can be expressed subjectively. Thus, subjective perception of quality of life is not always impaired by presence of disease, and any impact of disease on well-being is influenced by socio-demographic, psychological, social and environmental factors [2].

The internal reliability findings (inter-item and item-total correlations) verify the structural validity of the OIDP in both the Validation and Working Samples. All item-total correlations were above 0.20 as recommended for inclusion of an item in a scale. The standardised Cronbach's alpha value for the Working Sample was 0.71, lower than for the Validation Sample (0.79) but higher than for the original OIDP validation sample [6] and above the minimum value (0.70) recommended [27].

The lower Cronbach's alpha value in the Working Sample may be due to its larger size and the logarithmic distribution of OIDP scores. All diagnostic instruments show lesser consistency and reliability when used in populations with a lower prevalence or severity of events. The Cronbach's alpha varies as a function of the scale score in a given population. Thus, the alpha value was higher in the Validation Sample, which reported a higher impact level in all dimensions, but this does not imply that the instrument would be less valid in populations with a lower impact level, such as the Working Sample. In fact, the instrument served to classify the Working Sample as a population with lower oral impact.

Although the total OIDP scores were skewed to the left, all the analyses give the mean value, because more than half of the Working Sample scored zero and the median value therefore lost relevant information.

We have ensured the suitability of the dimensional framework of the OIDP for our target population by confirming that covered all aspects most highly valued by participants (appearance, eating, cleaning...). The "Working dimension" was also ratified in the Working Sample, with 62.5% of participants considering that their mouth influenced their occupational performance, mainly due to dental pain. We draw attention to the intermediate impacts collected (Table 5), since "Susceptibility" to oral disease was found to be a prevalent concern in this low-disease population. This concept does not strictly constitute an intermediate impact (see Methods), but was felt to be a consequence of past oral impairments and emerged in response to the same question on complaints about the mouth.

Modulating factors are depicted in Table 3 and Table 6. From a socio-demographic view the sex of the individual showed a major influence on both indicators i.e. women are more disabled and less satisfied with mouth as reported other authors [32,33]. About behavioural factors, hygiene level and time spent since last visit to the dentist were positively correlated with satisfaction and impacts levels respectively, in accordance with other authors [34,35]. From clinical perspective the presence of decayed teeth, the need for extraction or endodontic treatment and above all their location in the visible area (premolars, canines or incisors) demonstrated significant association with the impact and satisfaction level. This is an important finding of this study, because this usual pain-related condition could impact even stronger when decayed teeth are visible. Visible teeth have an important role in social interactions and this may become the primary function of the mouth in populations very concerned about appearance [36]. Prosthodontic variables mainly influenced the satisfaction rather than the impact level (Eichner Index, occlusal units, number of replaceable functional teeth). These factors have been previously pointed out as predictors of oral well-being [11,35-37]. This finding implies that prosthodontic variables are stronger predictors of satisfaction than oral impacts, and subjects could perform well in several daily activities (OIDP) without being satisfied with their mouth, because as it was hypothesized, satisfaction could also be affected by values, beliefs, expectations and self-comparisons with previous status. Periodontal variables representing a healthy state or an advanced disease accompanied by tooth mobility are coherently correlated with oral impacts and satisfaction [33,38].

Most of subjects were satisfied with their mouth (Table 2 and 5) in both samples. The prevalence of oral impact in both Validation (58.2%) and Working (46.0%) Samples can be considered moderate in comparison with previous findings using the OIDP [6-15,29-31,37]. Nevertheless, these prevalences are of concern since the OIDP is designed to solely measure "ultimate" impacts (disabilities or handicaps). Moreover, both samples exclusively comprised individuals who were not seeking dental treatment, and the evaluation period was only the previous six months. However this relative low floor effect (percentage of subjects with the lowest score) would be an appealing issue of the OIDP for using in longitudinal studies with dental patients since a global improvement of score is desirable to be detected. The difference in impact and satisfaction prevalences between samples (Table 8) would be explained by the social gradient in dental disease. This finding is similar to those observed in others studies using the OIDP [11,12,38]

The lowest prevalence of oral impact reported in an OIDP study was 13% in a British population of independent elderly individuals [10] and 18.3% in Norwegian adults [9]. A prevalence above 50% was described in OIDP studies of children [30], young people [13,14], adults [16,31] and elderly [7,8,11,37]. All of these studies identified the eating dimension as the most frequently affected. The prevalence of impacts related to eating in the Validation and Working Sample (38.3% and 23.5%) were above those reported by adults in Norway [9] (11.3%), but similar to Persians (35.1%) or Greeks (29.9%). This would be in line with the discrepancies in oral health status and cultural values of mouth between populations.

As found in all oral health quality of life studies, the main negative factor for oral well-being was dental pain ("impact value"). Important findings of the present study included the "impact *extension*" of "Oral ulcers", which affected all dimensions, and third-molar disease, which affected all dimensions except "Smiling". We also highlight the "impact prominence" of "bad breath" in the "Social dimension" and of "TMJ pain- dysfunction" in the "Sleeping and Relaxing" dimension. Research into the causes of impact has given less importance to these variables because most OIDP studies [7,8,10-12,37] have been in elderly populations, explaining the predominance in the literature of the effects of tooth pain, poorly fitting prostheses and edentulism.

The OIDP allows the cause of impacts to be linked with the affected dimension by means of the Condition-specific OIDP (CS-OIDP). This is a coded battery of 20 potential causes of impacts, from which the participant selects the cause(s) of specific difficulties. However, we employed an alternative method of reporting and describing the causes of oral impacts: causes were first reported by subjects, then confirmed by clinical examination and finally recorded. This is because an individual may report, for example, dental pain (toothache), when the specific cause is a broken tooth, decay, swollen gum, tooth sensitivity or loose tooth, distinct CS-OIDP categories with widely differing therapeutic options and costs. Thus, the presence of third molar pain implies that the tooth cannot be conserved by restorative dentistry and that extraction is required to alleviate the pain. A further reason for using an alternative approach is that the CS-OIDP battery does not contain some causal entities with high "impact value", such as Temporomandibular Pain Dysfunction Syndrome, third-molar or prosthetic problems. Another feature of our method is that when more than one causal entity affects an activity, individuals must use their judgement to select the most severe one, for which frequency and period variables will be recorded. This approach increases the value of each cause within dimensions, and could be used by planners to prioritise care and resources according to the impact descriptors. However, although the overscoring of some minor causes is avoided by this means, some relevant causes of impact may be underscored or even lost. Our approach differs from the original CS-OIDP but has the same objective (to optimise the assessment of dental treatment need) and uses the same tool (OIDP) and construct, i.e., three levels of impact.

Some existing impact descriptors have been successfully used to give a simpler description of affected dimensions ("extension") and to differentiate between individuals with several minor impacts and those with few but very severe impacts ("intensity") [30]. These descriptors largely address dimension involvement or performance scores rather than the behaviour of causes and predominance. In the present study, "value" and "prominence" were used alongside "extension" to explore in greater depth the behaviour of each cause among dimensions. These data could be used to compare causes among dimensions between and within populations, because it is possible that the predominance of different dimensions varies across sociocultural backgrounds and over the lifespan, emphasizing the effects of some specific conditions. Moreover, the "value" and "prominence" of the entities would allow to create a specific version of OIDP to explore the impact of specific causes with either low (e.g. trigeminal neuralgia, paresthesia) or high (e.g., removable prosthesis, orthodontic appliances) frequency by dividing the affected dimensions into subscales and exploring new impact-related dimensions that are only relevant for the clinical condition in question.

The main disadvantage of the OIDP is that it cannot be self-completed and requires a calibrated interviewer to apply it, whereas the OSS is very simple to use and is able to measure both good and poor well-being rather than just poor, although relevant information is lost because it is a unidimensional scale.

As it was hypothesized the socio-demographic profile of populations influenced directly and indirectly (values, behaviours...) the oral status, and it is the primary determinant of some health perceptions (complaints and intermediate impacts) that really affect oral satisfaction (OSS) and daily performances (OIDP). An immediate consequence of the oral impacts and satisfaction would be the perceived dental treatment needs. We believe that a complete evaluation of the oral health-related quality of life requires an assessment of oral satisfaction, not merely confirmation of absence oral impacts. Exploring simultaneously these issues may provide useful insights into how satisfaction and impact on well-being are constructed.

Descriptive research would support the development of hypotheses to be tested in well-conducted studies. Longitudinal studies are required to examine the sensitivity of these indicators to detect changes in oral well-being after therapeutic interventions. Future studies should analyse why these mostly satisfied populations were mostly affected by complaints, treatment need and oral impacts. The adaptation to some impacts, complaints or perceived need should be a part of the human resistance.

### Limitations of the study

In the present study the OIDP was not analysed in terms of Test-retest reliability and its reliability was solely studied by means of internal consistency and validation tests. The results of this study might not be representative of the Spanish population, because in the Validation Sample there was a bias towards low socio-occupational groups (e.g., pensioners and unemployed) and all subjects in the Working Sample were healthy active Regional Government officers (bias towards high socio-occupational groups). However, we consider that Working Sample could represent the adult working population in Spain, since their socio-demographic and clinical characteristics match those reported for this age range in the most recent (2005) National Oral Health Survey [39], and the sample size (n = 561) was adequate. The cross-sectional design adopted in this study, although indicated for questionnaire validation, reduces the level of evidence of the associations reported that should be interpreted with caution.

## Conclusion

The OIDP and OSS are reliable and valid indicators of oral impacts and oral satisfaction respectively in an adult Spanish population. Exploring simultaneously these issues may provide useful insights into how satisfaction and impact on well-being are constructed.

Oral health-related quality of life is determined by: socio-demographic factors, e.g gender; behavioural variables e.g. hygiene level and time since last visit to dentist; clinical factors, e.g., deep caries (endodontic or exodontic), especially in the visible area. Prosthodontic factors mainly influence oral satisfaction rather than impact level, and periodontal factors have no effect on either satisfaction or impact unless the disease is in an advanced stage with dental mobility.

## Abbreviations

OIDP: Oral Impacts on Daily Performances; OSS: Oral Satisfaction Scale; OHRQoL: Oral Health-Related Quality of Life; WHO: World Health Organization; DMFT: Decayed, missing and filled teeth; TMJ: Temporomandibular junction.

## Competing interests

The authors declare that they have no competing interests.

## Authors' contributions

MB conceived and coordinated the study from its design to the manuscript confection. JM carried out the study and drafted the manuscript. AA made contributions to the conception, design, data analysis and interpretation. All authors read and approved the final manuscript.
